# Identification of virulence factors and antibiotic resistance in *Salmonella enterica* subsp. enterica serovar Javiana (FARPER-220) isolated from broiler chickens

**DOI:** 10.1128/MRA.00327-23

**Published:** 2023-11-09

**Authors:** Luis Tataje-Lavanda, Doris Villanueva-Pérez, Angela Montalván-Avalos, Katherine Vallejos-Sánchez, Mirko Zimic-Peralta, Manolo Fernández-Sánchez, Manolo Fernández-Díaz

**Affiliations:** 1 Laboratorios de Investigación y Desarrollo, FARVET, Chincha Alta, Ica, Peru; 2 Escuela Profesional de Medicina Humana, Universidad Privada San Juan Bautista, Lima, Peru; 3 Laboratorio de Bioinformática, Biología Molecular y Desarrollos Tecnológicos. Laboratorios de Investigación y Desarrollo, Facultad de Ciencias, Universidad Peruana Cayetano Heredia, Lima, Peru; University of Maryland School of Medicine, Baltimore, Maryland, USA

**Keywords:** *Salmonella *Javiana, virulence factors, antibiotic resistance, genome sequencing, broiler chickens

## Abstract

Genome sequencing of highly virulent *Salmonella enterica* subsp. enterica serovar Javiana strain FARPER-220 (ST-1674) isolated from broiler chickens in Peru revealed multiple virulence factors, antibiotic resistance genes, and invasion-related subcategories. The results provide insights into the potential importance of this strain in causing infections in various animals.

## ANNOUNCEMENT

*Salmonella enterica* subsp. enterica serovar Javiana (*S.* Javiana) is a highly virulent bacterium that infects various animals, including birds (1). It belongs to the family Enterobacteriaceae and serogroup D1 (O:9) and can cause systemic infections in susceptible individuals. *S.* Javiana produces cytotoxic distending toxin or typhoid toxin; this toxin plays a crucial role in DNA damage and systemic colonization of the host (2).

The FARPER-220 strain was isolated from a homogenized mixture of liver and spleen of broiler chickens in southern Peru in 2017. The strain was identified as *S. enterica* through colony morphology and specific PCR (3). Genomic DNA was extracted from an overnight culture grown in Brain Heart Infusion broth at 37°C using a phenol-chloroform protocol (4). Antibiograms were performed by the Kirby-Bauer disk diffusion method (Table 1) following CLSI standards (5).

**TABLE 1:** Phenotypic resistance of FARPER-220^*a*^

Antibiotic	Concentration (µg)	Result
Amoxicillin/clavulanic acid	30	Sensitive
Ampicillin	10	Sensitive
Colistin	10	Resistant
Doxycycline	30	Resistant
Enrofloxacin	5	Sensitive
Florfenicol	30	Resistant
Fosfomycin	50	Resistant
Gentamicin	10	Resistant
Lincomycin	2	Resistant
Neomycin	30	Intermediate
Oxacillin	1	Resistant
Penicillin	10	Resistant
Streptomycin	300	Sensitive
Sulfamethoxazole/trimethoprim	25	Resistant
Tetracycline	30	Sensitive

^*a*^ Kirby-Bauer disc diffusion method antibiogram results following CLSI standards (5).

Whole-genome sequencing of FARPER-220 was performed with a 20-kb SMRTbell library (PacBio DNA/polymerase binding kit P6) on the PacBio RS II platform (PacBio DNA sequencing kit 4.0) using C4 chemistry with eight single-molecule real-time (SMRT) cells. The genome was assembled *de novo* using RS HGAP v.3.0 (6) on SMRT Portal (v.2.3) by default (Macrogen, Inc., South Korea). The genome circularization was performed manually, aligning the ends of linear complemented genome segments to form the chitinase-encoding gene.

We used default parameters for all software unless specified otherwise. Genomic annotation was executed utilizing the NCBI Prokaryotic Genomic Annotation Pipeline v.4.8 (7) and RASTtk (8) for functional analysis. Strain classification was corroborated through KmerFinder v.3.2 (9, 10), sistr_cmd (Galaxy Version 1.1.1+galaxy1) (-p -n –use-full-cgMLST-db -m --qc) (11), ANI Calculator v.1 (12), and BLASTn (13) against the nr/nt database. Resistant genes were identified using ResFinder v.4.3.3 (14).

A total of 87,566 subreads passed filtering (Default) were generated, with an average length of 7,075 bp and an N50 of 11,086 bp. The assembled genome resulted in one closed circular chromosome of 4.6 Mbp (%GC: 52.2; depth coverage: 99×) and 4,649 genes identified (4,529 coding sequences [CDS] and 120 RNA). *In silico* identification by average nucleotide identity (ANI) revealed that FARPER-220 has the closest genome complete to *S.* Javiana CFSAN001992 (CP004027.1) (15) (99.91% identity) and CFSAN079101 (CP042442.1) (99.59% Identity) isolated in the USA. Serovar determination, conducted through Mash analysis, identified FARPER-220 as belonging to serovar Javiana within serogroup D1.

The gene *acc(6′)-Iaa* (aminoglycoside resistance), which confers resistance to Amikacin and Tobramycin, was identified with full gene coverage and 96.8% identity against the ResFinder database (NC_003197). Moreover, BLAST showed 100% cover and identity of the gene *acc(6′)-Iaa* with FDAARGOS_707 (CP046279.1), FDAARGOS_688 from Nigeria (CP046280.1), CFSAN023357 (CP086351.1), and CFSAN023356 from the USA (CP086353.1).

Functional annotation, complemented by RASTtk analysis, predicts numerous subcategories associated with antibiotic and toxic compound resistance. These encompassed the mercury-resistance operon, copper homeostasis, cobalt-zinc-cadmium resistance, fluoroquinolone resistance, copper tolerance, adaptation to d-cysteine, zinc resistance, and mercuric reductase. Furthermore, functional subcategories indicative of invasion and intracellular resistance were identified, notably the Mycobacterium virulence operon implicated in protein synthesis (SSU ribosomal proteins), DNA transcription, and potential involvement in quinolinate biosynthesis.

In conclusion, the complete genome sequence of *S*. Javiana, isolated from broiler chickens in Peru, has been identified, as well as various virulence factors and antibiotic-resistance genes.

## Data Availability

The complete genome was deposited in GenBank under the accession number CP038233.1. Raw sequence reads were deposited under the accession number SRR8903084.
